# Vitamin D status in North American managed black rhinoceros (*Diceros bicornis*) and white rhinoceros (*Ceratotherium simum*)

**DOI:** 10.1093/conphys/coag062

**Published:** 2026-08-25

**Authors:** Shauni Windle, Terri L Roth

**Affiliations:** Center for Conservation and Research of Endangered Wildlife (CREW), Cincinnati Zoo & Botanical Garden, 3400 Vine Street, Cincinnati, OH 45220, USA; Center for Conservation and Research of Endangered Wildlife (CREW), Cincinnati Zoo & Botanical Garden, 3400 Vine Street, Cincinnati, OH 45220, USA

**Keywords:** 25-Hydroxyvitamin d (25ohd), cholecalciferol, ergocalciferol, rhinoceros reproductive status, solar irradiance zone, ultraviolet B radiation and vitamin D

## Abstract

Black rhinoceros (*Diceros bicornis*) and white rhinoceros (*Ceratotherium simum*) in managed care experience environmental conditions that differ from those in the wild, including potential variation in exposure to ultraviolet B radiation, which may influence vitamin D status. In other species, vitamin D insufficiency has been linked to skeletal, muscular, and reproductive disorders, but information on vitamin D in managed rhinoceros remains limited. In this study, liquid chromatography–tandem mass spectrometry (LC–MS/MS) was used to quantify serum 25-hydroxyvitamin D_2_ and 25-hydroxyvitamin D_3_ (25OHD_2_ and 25OHD_3_, respectively) concentrations in black and white rhinoceroses housed at North American institutions. Data were evaluated for potential differences in vitamin D associated with species, sex, age, season, location, and female reproductive status. 25OHD_2_ was undetectable in more than 50% of white rhinoceros samples and 80% of black rhinoceros samples. Conversely, 25OHD_3_ concentrations varied significantly with season and location (*P* < 0.05), with higher concentrations during summer months and in regions of greater solar irradiance. No relationships were identified between 25OHD_3_ concentrations and species, sex, age, or reproductive status. These findings provide the first vitamin D reference data for managed white rhinoceros and expand available information for black rhinoceroses. Together, the results suggest that environmental factors such as sunlight exposure have a greater influence on circulating vitamin D than demographic characteristics, providing a foundation for further investigation into how management conditions impact vitamin D metabolism and associated health in managed rhinoceroses.

## Abbreviations

25ohd: 25-hydroxyvitamin d

## Introduction

Conservation research and managed care programmes have made substantial advances in meeting the needs of animals in *ex situ* care. Diets are formulated to address nutritional requirements, habitats are designed to promote species-specific behaviours, and preventative health measures continue to improve (Ricketts *et al.*, 2020; de Azevedo *et al.*, 2023; Martelli and Krishnasamy, 2023; Fens and Clauss, 2024). Despite this progress, some elements of native ranges, including environmental conditions, cannot be fully replicated due to geographic and climatic constraints (Liu *et al.*, 2017; Doyle *et al.*, 2024). These limitations may impact the welfare and health of zoo-managed species, including black (*Diceros bicornis*) and white (*Ceratotherium simum*) rhinoceroses.

One such environmentally dependent factor that is difficult to replicate in managed settings is vitamin D provision, including its two precursor forms, ergocalciferol and cholecalciferol. Ergocalciferol is derived from plant material via ultraviolet B (UVB)-irradiated microorganisms (e.g. fungi) on plants, whereas cholecalciferol is primarily synthesized in the skin upon UVB exposure, although limited dietary sources (e.g. fish and a small number of plant species) may provide small quantities (Boland *et al.*, 2003; Jäpelt *et al.*, 2011). Following absorption, both precursor forms are converted in the liver to 25-hydroxyvitamin D_2_ and 25-hydroxyvitamin D_3_ (25OHD_2_ and 25OHD_3_), which serve as the circulating reservoir and are the forms most commonly measured in clinical assays (Dirks *et al.*, 2018). Each 25OHD form is subsequently converted in the kidneys to the respective bioactive form, 1,25-dihydroxyvitamin D (1,25OHD_2_). The bioactive forms support several physiological processes, particularly those related to calcium absorption and immune function, including bone density retention, muscle control, and reproduction (Bikle, 2012; Ceglia and Harris, 2012; Coffey *et al.*, 2012; Handel *et al.*, 2016; Grzesiak *et al.*, 2022).

The primary form of vitamin D utilized differs among species. There is evidence that equids rely predominantly on diet-derived ergocalciferol (Dosi *et al.*, 2023), whereas caprids depend on induced UVB-induced cutaneous production of cholecalciferol (Handel *et al.*, 2016). Regardless, for many domestic and zoo-managed mammals, vitamin D status and the health consequences of deficiency or toxicity remain poorly understood (Hymøller and Jensen, 2015; Hurst *et al.*, 2020; Liptovszky *et al.*, 2025). In the limited published data on vitamin D status in zoo mammals, reported conditions linked with suspected vitamin D deficiency include metabolic bone disease in flying foxes (*Pteropus* spp.) and pied tamarins (*Saguinus bicolor*), as well as cardiovascular disease in chimpanzees (*Pan troglodytes*) (Lopez *et al.*, 2001; Faim *et al.*, 2025; Liptovszky *et al.*, 2025). Although such vitamin D deficiency-related disorders are not reported in managed rhinoceroses, immune (Nelson *et al.*, 2012; Xu *et al.*, 2018) and reproductive dysfunction are recognized areas of concern in this taxon (Corder *et al.*, 2023; Roth, 2024) and have been linked to vitamin D status in other species (Moittié *et al.*, 2022). Given that the native ranges of black and white rhinoceroses in Africa receive substantially higher UVB radiation than regions in Europe and North America, vitamin D supplementation is often used in managed settings to offset reduced environmental UVB exposure (Olds *et al.*, 2018; Kift and Webb, 2024). However, vitamin D status in managed populations remains poorly defined, highlighting the need for reference values.

Successful reproduction in managed rhinoceroses is critical for long-term species conservation (Roth, 2024). Proposed factors contributing to reduced reproductive success include reproductive pathologies, obesity-associated dysfunction, exogenous endocrine disruption, and social influences (Hermes *et al.*, 2006; Edwards *et al.*, 2015; Williams *et al.*, 2019; Schwarzenberger and Pannrucker, 2023). However, direct cause and effect has been difficult to prove, and it is unlikely that a single factor fully explains observed reproductive challenges. Lower circulating vitamin D concentrations may represent an additional, underexplored contributor. In domestic and wild animal species, higher vitamin D concentrations are related to improved reproductive outcomes, including increased fecundity in wild sheep, oestrus induction in horses, oestradiol production and folliculogenesis in mice, and milk yield in cattle (Kinuta *et al.*, 2000; Handel *et al.*, 2016; Zhou *et al.*, 2019; Bukhari and Urooj, 2025). Collectively, these findings support a role for vitamin D in reproductive physiology across taxa.

Vitamin D data in rhinoceroses are limited with respect to both species representation and differentiation between vitamin D forms. Most published studies report total 25OHD concentrations without distinguishing between 25OHD_2_ and 25OHD_3_ and/or are restricted to black and greater one-horned (GOH; *Rhinoceros unicornis*) rhinoceroses (Clauss *et al.*, 2002; Bapodra *et al.*, 2014; Olds *et al.*, 2018; Young *et al.*, 2019, unpublished work; Bruins-van Sonsbeek and Corbee, 2024; Bruins-van Sonsbeek *et al.*, 2025) (Table 1). Expanding analyses to differentiate circulating vitamin D across a large population of North American managed black and white rhinoceroses may provide essential reference values for these taxa. Additionally, evaluating how demographic characteristics relate to circulating vitamin D may provide further context for interpreting vitamin D status. Such a dataset would offer a foundation for assessing the relevance of vitamin D to reproduction and health in North American rhinoceros populations.

**Table 1:** Vitamin D concentrations (ng/ml; ±SD) reported for wild and zoo-managed black rhinoceroses (*Diceros bicornis*), GOH rhinoceroses (*Rhinoceros unicornis*) and white rhinoceroses (*Ceratotherium simum*)

Rhinoceros species	Management	Assay	Season	*n*	25OHD	25OHD_3_	25OHD_2_
*D. bicornis*							
	Wild^a^	Protein-binding assay		28		55.7 ± 34.1	
	Managed^b^	LC–MS/MS	W	25		11.4 ± 9.76	1.92 ± 0.33
			S	28		20.7 ± 8.70	1.92 ± 0.26
	Managed^c^	LC–MS/MS	W	16		14.4 ± 13.3	
			S	20		23.9 ± 13.9	
	Managed^d^	RIA		2	40.2		
	Managed^e^	ELFA		5	50.6 ± 28.1		
	Managed^f^	ELFA		11	95.6 ± 108.2		
							
*R. unicornis*	Managed^g^	RIA	W	5	8.96 ± 1.17		
			S	5	13.1 ± 2.98		
	Managed^e^	ELFA		1	43.2		
							
*C. simum*	Managed^b^	LC–MS/MS	W	46		12.6 ± 6.60	2.51 ± 0.63
			S	47		23.2 ± 8.04	2.35 ± 0.76

Values were converted from their originally published units to ng/ml to match the units used in the present study. When available, means and SDs are presented by season (S—summer; W—winter) and assay type. Analytical methods include LC–MS/MS, protein-binding assay, RIA, and enzyme-linked fluorescent assay (ELFA). Sample size (*n*) values represent the number of sampled animals.

a ^a^ Clauss *et al.*, 2002.

b ^b^Present study.

c ^c^ Young *et al.*, 2019, unpublished work.

d ^d^ Olds *et al.*, 2018;

e ^e^ Bruins-van Sonsbeek and Corbee, 2024;

f ^f^ Bruins-van Sonsbeek *et al.*, 2025;

g ^g^ Bapodra *et al.*, 2014.

The aim of this study was to quantify 25OHD_2_ and 25OHD_3_ concentrations in a diverse population of North American rhinoceroses and to explore relationships with age, sex, species, and location. Vitamin D concentrations were also evaluated across reproductive states, with a focus on white rhinoceroses, given their history of reproductive challenges in managed populations (Roth, 2024).

## Materials and methods

### Animals and serum sampling

Paired winter and summer serum samples collected between 2022 and 2023 were obtained from the American Institute of Rhinoceros Science (AIRS) serum bank. Samples had been collected into additive-free, red-top tubes without gel separators, held at room temperature for 1 hour or refrigerated for 2–4 hours, and then centrifuged. Resulting serum was frozen and shipped to the Cincinnati Zoo & Botanical Garden’s Center for Conservation and Research of Endangered Wildlife (CREW), where samples were aliquoted and stored at −20°C. The dataset included 28 black rhinoceroses (13 female, 15 male; 4–32 years) and 47 white rhinoceroses (33 female, 14 male; 3–54 years).

For this project, sample pairs were selected based on collection month and the interval between seasonal samples. Winter samples were defined as those collected between November and January, and summer samples as those collected between May and July, reflecting seasonal daylight patterns and general management practises at North American institutions. To allow sufficient time for physiological changes in vitamin D status, paired samples were required to be separated by at least seven months (Olds *et al.*, 2018). Selected pairs were submitted to Heartland Assays (Ames, IA, USA) in August 2024. Vitamin D forms 25OHD_2_ and 25OHD_3_ were quantified using liquid chromatography–tandem mass spectrometry (LC–MS/MS), with a limit of quantification ranging between 1.5 and 200 ng/ml. Storage time, defined as the interval between serum collection and quantification assay, ranged from 0.8 to 2.4 years. Although storage time was not expected to influence 25OHD concentrations (Agborsangaya *et al.*, 2009), its relationship with 25OHD values was statistically evaluated.

### Ethical declaration

All serum samples were collected under Institutional Animal Care and Use Committee (IACUC) approval (#22–173) and under the discretion of the respective institutional veterinary and management teams.

### Solar irradiance zones

Samples were obtained from 27 institutions across 17 states. Solar irradiance zones (1–9) were assigned based on each institution’s location using the National Solar Radiation Database (Sengupta *et al.*, 2018). These zones reflect average daily solar irradiance from 1998 to 2016, with zone 1 receiving <4.00 kWh/m^2^/day and zone 9 receiving >5.75 kWh/m^2^/day.

### Reproductive status assignment

Reproductive states, including pregnancy (pregnant or non-pregnant), lactational (lactating or non-lactating), and reproductive activity (active or inactive), were determined at the time of sample collection. Pregnancy and lactational samples were identified by reviewing calf birth dates and then assigning samples collected 16 months prior to and 12 months following the date as classified as pregnant and lactating, respectively (Species360, 2024). Reproductive activity status was classified using pregnancy data and faecal progesterone metabolite (fPM) trends. Females categorized as reproductively active were either pregnant or displayed cyclic fPM fluctuations consistent with reproductive cyclicity, whereas inactive individuals exhibited static (i.e. within two standard deviations (SDs) of baseline), or persistently low fPM concentrations (i.e. flatliners) (Brown *et al.*, 2001).

### Statistical analyses

Statistical analyses were performed in R version 4.3.2 (R Core Team, 2024), using RStudio. Serum vitamin D concentrations were analysed using linear models when a single data point per rhinoceros was included (e.g. season-specific analyses), and linear mixed-effects models (package lme4; Bates *et al.*, 2015) were used when paired seasonal samples were available, with individual identity incorporated as a random effect. Solar irradiance zone and season were included as fixed factors in all mixed-effects models. Analyses were conducted separately by species, and sex-specific models were applied for reproductive comparisons. Species and sex effects were included as fixed factors, whereas age was evaluated as a continuous predictor. Zones represented by fewer than three individuals were excluded from analysis.

Faecal progesterone classifications of reproductive status in black rhinoceroses were limited to seven individuals, including one pregnant and two reproductively inactive females. Consequently, analyses of reproductive status were restricted to female white rhinoceroses (*n* = 27). Due to the potential influence of calcium demands during lactation (Wagner and Hollis, 2020), which may result in lower serum 25OHD values, reproductive comparisons were repeated without lactating females. Lactational status was visualized descriptively but not assessed statistically due to the small number of individuals and limited representation. Statistical significance was defined as *P* < 0.05 for all analyses.

25OHD_3_ reference intervals (RIs) were determined separately for each rhinoceros species and season, following the guidelines provided by the American Society for Veterinary Clinical Pathology (ASVCP; Friedrichs *et al.*, 2012), and using a non-parametric percentile method. Each animal contributed a single observation per season. Because 25OHD_3_ concentrations were not assumed to follow a Gaussian distribution, the RI was defined as the 2.5th and 97.5th percentiles of the observed values within each species-season group, and 90% bootstrap confidence intervals (CIs) were calculated for the lower and upper reference limits using 1000 resamples. Median, observed minimum and maximum, and the RI were reported for descriptive purposes (Table 2). Negative values were not biologically possible, and lower bounds were constrained to zero where necessary. Shapiro–Wilk testing was performed only as an exploratory assessment of distributional shape and was not used to determine the analytic method.

**Table 2:** Reference interval (RI) statistics for serum 25-hydroxyvitamin D_3_ values (ng/ml) of zoo-managed black rhinoceroses (*Diceros bicornis*) and white rhinoceroses (*Ceratotherium simum*) in North America

Rhinoceros species and season	*n*	Median	RI	Lower 90% CI	Upper 90% CI
*D. bicornis*					
Winter	25	21.8	6.0–35.7	5.8–9.7	30.0–42.9
Summer	28	8.6	1.5–32.1	1.5–2.4	25.7–35.5

*C. simum*					
Winter	46	21.7	11.1–39.8	9.4–14.6	33.6–52.6
Summer	47	13.5	2.1–27.1	1.3–3.3	19.1–31.5

CI and RI values were quantified following the guidelines provided by the ASVCP (Friedrichs *et al.*, 2012).

These RIs should be interpreted with caution because sample sizes were modest, individual health histories were unavailable, and pregnant or lactating individuals were retained to avoid further reducing already small sample sizes; therefore, the reported RIs are best viewed as practical species-season summaries rather than definitive clinical reference limits.

## Results

### Vitamin D assay

Undetectable 25OHD_2_ concentrations represented more than 50% of white rhinoceroses and more than 80% of black rhinoceroses samples. Among samples with detectable 25OHD_2_ concentrations, summer values ranged from 1.6 to 2.2 ng/ml in black rhinoceroses and 1.5 to 3.8 ng/ml in white rhinoceroses, and winter concentrations ranged from 1.6 to 2.4 ng/ml in black rhinoceroses and 1.5 to 4.0 ng/ml in white rhinoceroses. Due to the high proportion of samples below assay detection limits and the limited number of individuals with detectable values in both of their samples, 25OHD_2_ was excluded from statistical analyses for both species.

In contrast, 25OHD_3_ was detectable in all but two winter black rhinoceroses samples (*n* = 28) and one winter white rhinoceroses sample (*n* = 47). Summer 25OHD_3_ concentrations ranged from 6.1 to 42.9 ng/ml in black rhinoceroses and 9.4 to 52.6 ng/ml in white rhinoceroses, whereas winter concentrations ranged from 2.1 to 35.5 ng/ml in black rhinoceroses and 3.1 to 31.5 ng/ml in white rhinoceroses. Storage time did not influence 25OHD_3_ concentrations for either species (*P >* 0.05).

### Effect of season and location on vitamin D concentrations

**Figure 1:**
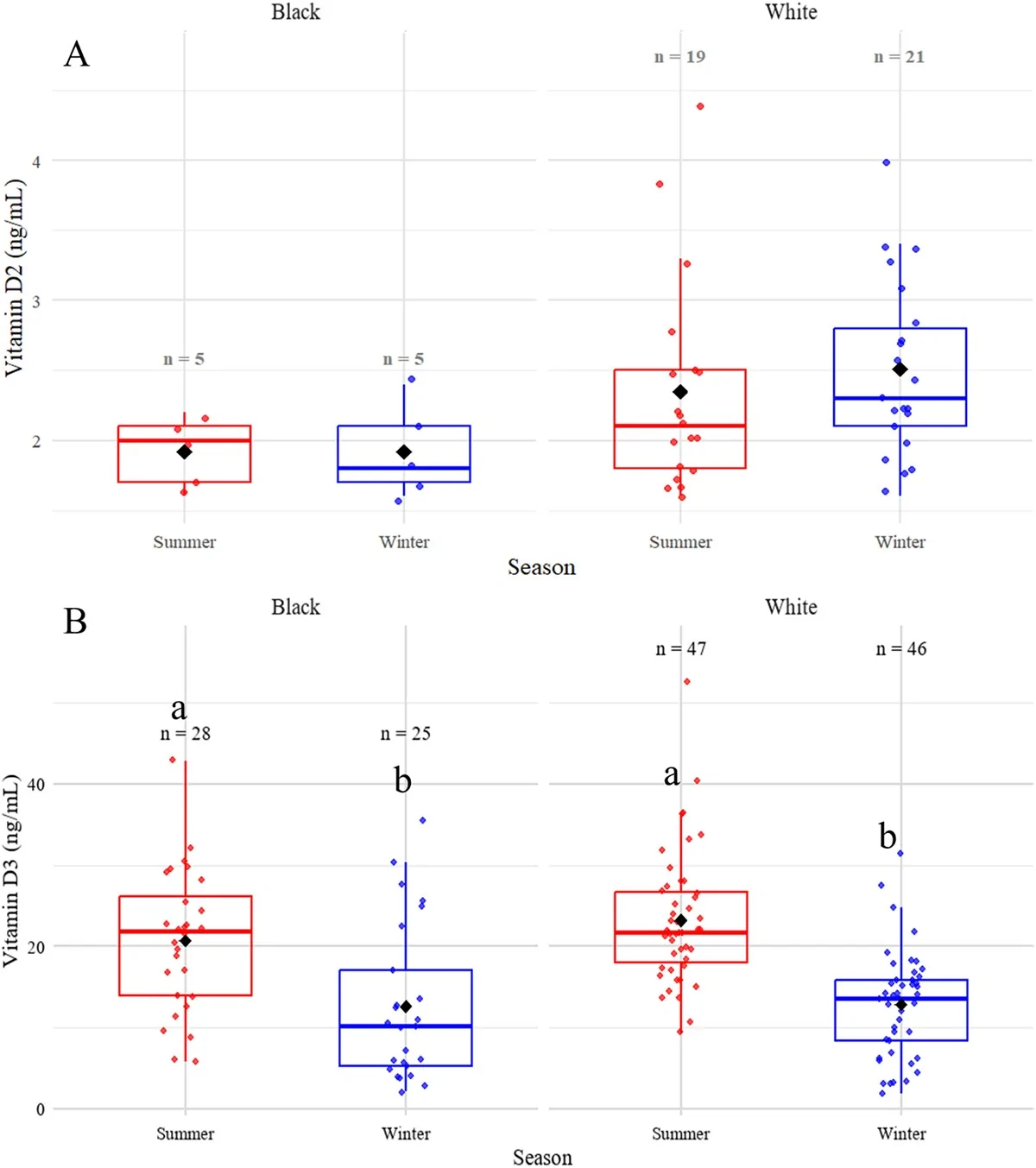
Circulating (**A**) 25OHD_2_ and (**B**) 25OHD_3_ concentrations in black rhinoceroses (*Diceros bicornis*) and white rhinoceroses (*Ceratotherium simum*) by season. Serum vitamin D values were quantified using LC–MS/MS. Paired serum samples were categorized by collection season: summer (May–July) and winter (November–January). Mean vitamin D values are indicated by diamonds (◊). Statistical analyses were not performed for 25OHD_2_ due to low sample numbers. Mean 25OHD_3_ values were significantly higher in the summer than winter for both species, as indicated by lettered superscripts (*P* < 0.05). No species differences were detected (*P* > 0.05). A linear mixed-effects model was used, with season, species, solar irradiance zone, and individual included as fixed effects.

**Figure 2:**
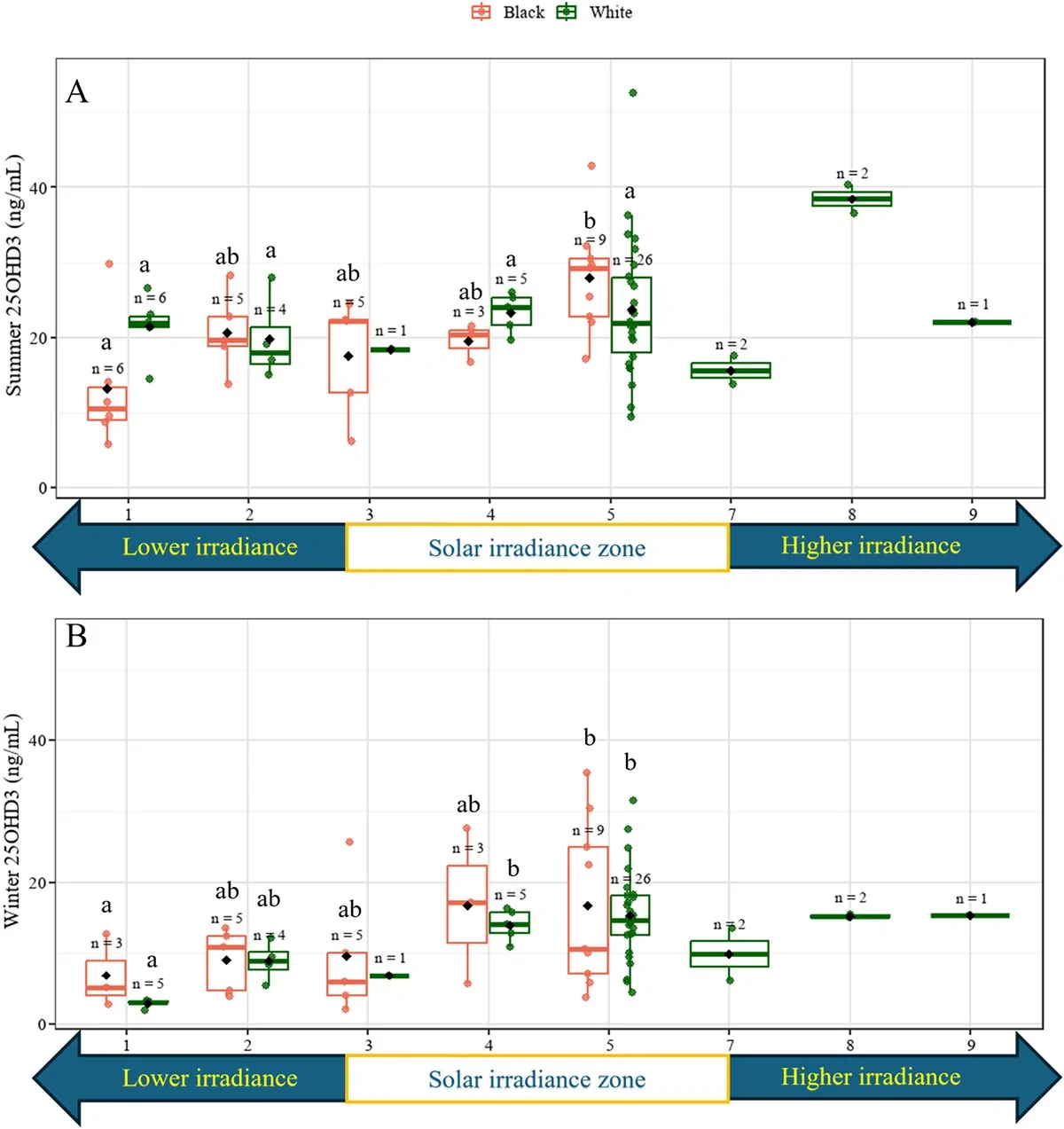
Circulating 25OHD_3_ of white rhinoceroses (*Ceratotherium simum*; green boxes) and black rhinoceroses (*Diceros bicornis*; orange boxes) by solar irradiance zone and season. Individual rhinoceroses were assigned to specific zones based on the location of the home institution, as identified by the National Solar Radiation Database (Sengupta *et al.*, 2018). Mean 25OHD_3_ values are indicated by diamonds (◊), and values with different superscripts within each species differed significantly (*P* < 0.05). Statistical analyses were limited to zones with three or more values. Data were analysed using a linear mixed-effects model with solar irradiance zone, season, and individual included as fixed effects.

Circulating 25OHD_3_ concentrations were significantly greater in summer than in winter for both species (*P* < 0.0001 for each; Fig. 1), with average concentrations declining by approximately two-fold from summer to winter. Concentrations also varied by solar irradiance zone, but analyses were constrained by the uneven distribution of individuals across zones. Accordingly, analyses were restricted to zones represented by at least three individuals within each species, resulting in four qualifying zones for white rhinoceroses and five for black rhinoceroses (Fig. 2). Within these zones, 25OHD_3_ concentrations varied with both solar irradiance zone and season in each species.

In white rhinoceroses, a significant interaction between zone and season (*P* = 0.008) indicated that the magnitude of seasonal differences was influenced by zone. During winter, 25OHD_3_ concentrations in zone 1 (<4.00 kWh/m^2^/day) were significantly lower than those observed in zone 4 (4.50–4.75 kWh/m^2^/day; *P* = 0.035) and zone 5 (4.75–5.00 kWh/m^2^/day; *P* = 0.001). No differences among zones were detected during the summer.

In black rhinoceroses, 25OHD_3_ concentrations differed by solar irradiance zone (*P* = 0.019) and season (*P* < 0.001); however, no interaction between zone and season was detected (*P* = 0.57). Mean 25OHD_3_ concentrations increased with higher solar irradiance zones, with individuals in zone 5 exhibiting higher concentrations than those in zone 1 in both seasons (*P* = 0.010).

### Effect of species, age and sex on vitamin D concentrations

**Figure 3:**
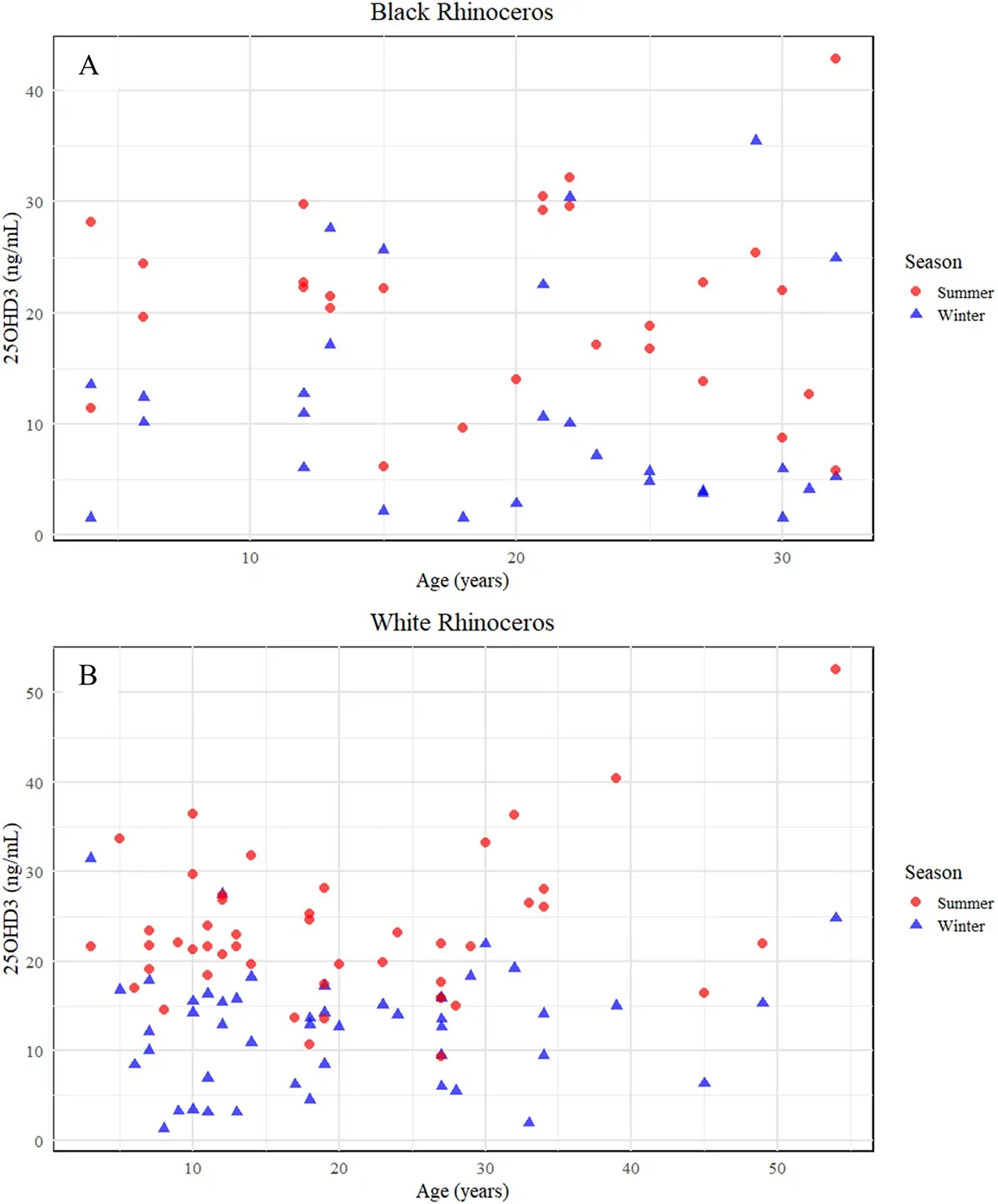
Circulating 25OHD_3_ of (**A**) black rhinoceroses (*Diceros bicornis*; *n* = 28) and (**B**) white rhinoceroses (*Ceratotherium simum*; *n* = 47) by age. Age did not significantly influence circulating 25OHD_3_ in either black (*P* = 0.06) or white (*P* = 0.47) rhinoceroses. Data were analysed using a linear mixed-effects model with solar irradiance zone, season, and individual included as fixed effects.

No differences in 25OHD_3_ concentrations were detected between white and black rhinoceroses when accounting for season and solar irradiance zone. Across both species, 25OHD_3_ concentrations did not differ with age (Fig. 3; white *P* = 0.39; black *P* = 0.39) or sex (white *P* = 0.20; black *P* = 0.85).

### Association of vitamin D concentrations and reproduction

**Figure 4:**
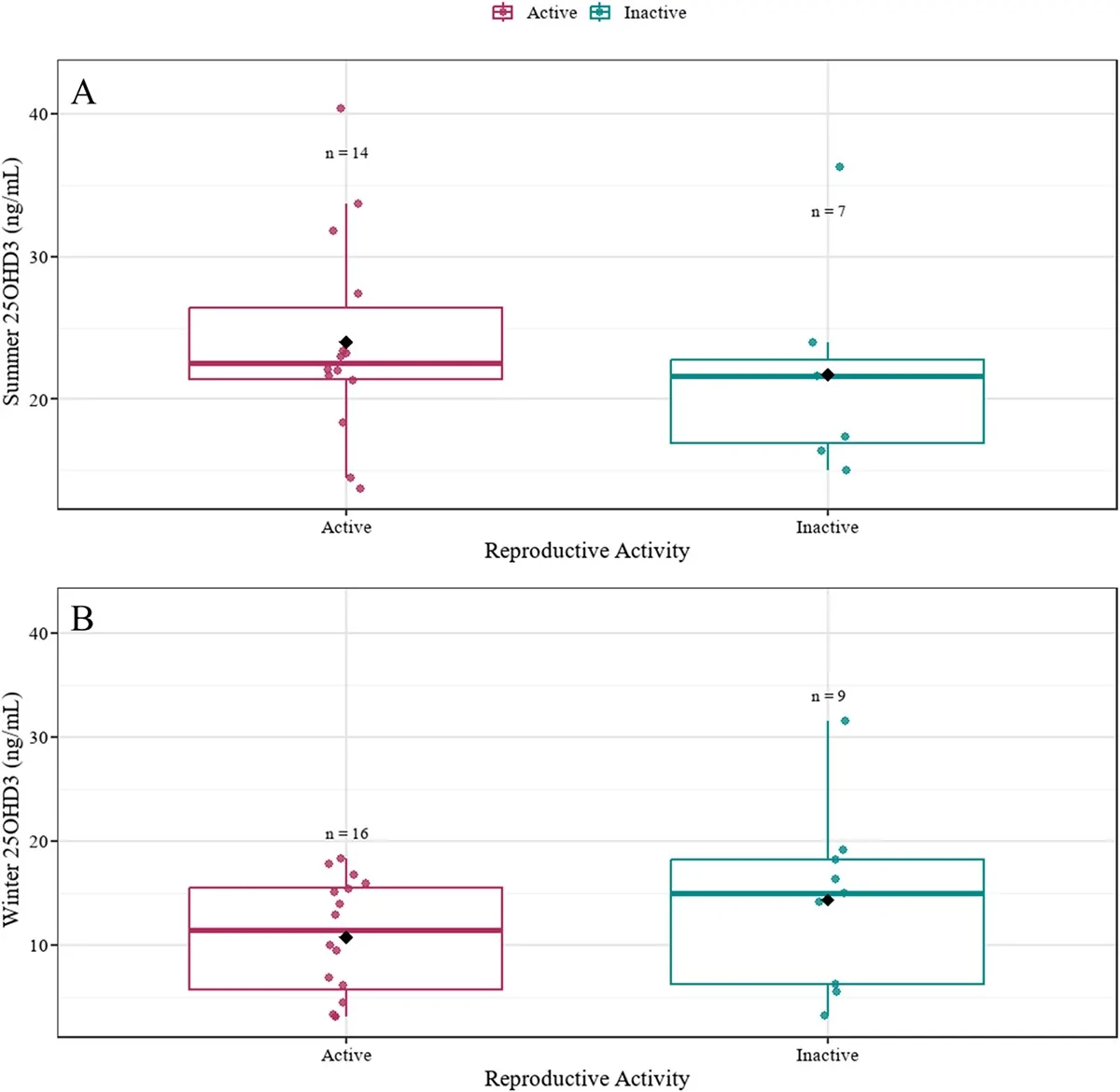
Circulating 25OHD_3_ concentrations in white rhinoceroses (*Ceratotherium simum*) by season (**A**—Summer; **B**—Winter) and reproductive activity status. Reproductive activity was determined by fPM patterns. Individuals classified as reproductively active exhibited fPM fluctuations indicative of ovarian activity or pregnancy during the time of sampling, whereas inactive individuals exhibited fPM trends that remained below baseline or failed to rise more than two standard deviations above baseline. Mean 25OHD_3_ values are indicated by diamonds (◊). No significant relationship was detected between 25OHD_3_ concentrations and reproductive activity status (*P >* 0.05). Data were analysed using a linear mixed-effects model with solar irradiance zone, season, and individual included as fixed effects.

**Figure 5:**
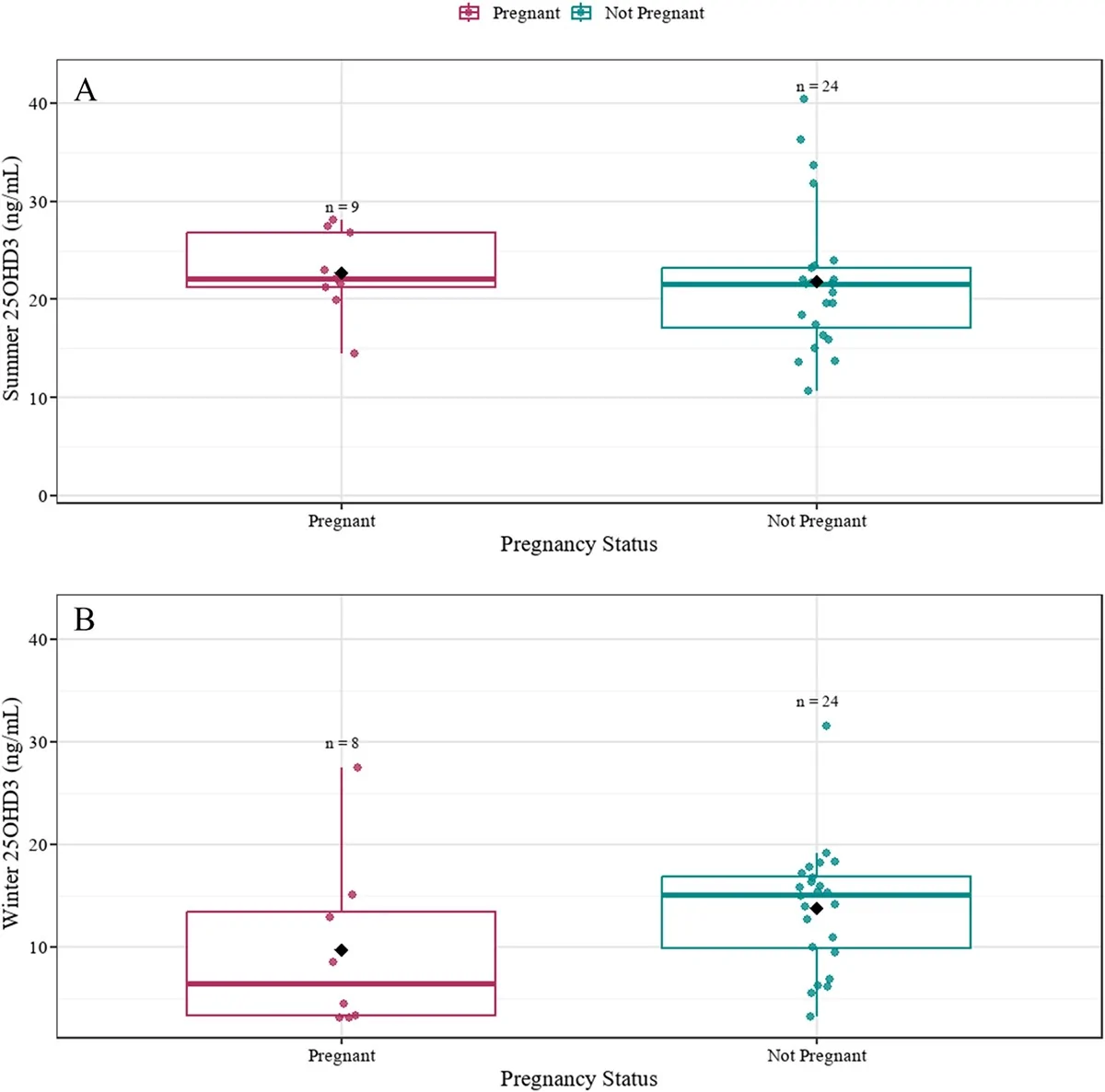
Circulating 25OHD_3_ concentrations in white rhinoceroses (*Ceratotherium simum*) by season (**A**—Summer; **B**—Winter) and pregnancy status. Mean 25OHD_3_ values are indicated by diamonds (◊). Pregnancy status was provided by institutions and confirmed by review of calving dates in the Zoological Information Management database (Species360, 2024). No significant relationship was detected between 25OHD_3_ concentrations and pregnancy status (*P >* 0.05). A linear mixed-effects model was used for analysis with solar irradiance zone, season, and individual included as fixed effects.

Concentrations of 25OHD_3_ did not vary significantly with white rhinoceroses reproductive cyclicity (*P* = 0.92; Fig. 4) or pregnancy status (*P* = 0.31; Fig. 5). Exclusion of lactating individuals did not alter the results for either reproductive activity (*P* = 0.93) or pregnancy (*P* = 0.19) analyses. Descriptively, mean 25OHD_3_ concentrations from lactating females (*n* = 3 in winter; *n* = 7 in summer) and non-lactating females (*n* = 9) differed by less than 5 ng/ml within the same institution in both seasons (Fig. S1).

## Discussion

In this study, vitamin D was quantified in black and white rhinoceroses across North American institutions, and relationships with location and demographic characteristics, including species, sex, age, and reproductive status, were explored. Our results provide important reference values for North American populations and demonstrate fluctuations in serum 25OHD_3_ across solar irradiance zones and seasons. Furthermore, the absence of associations between 25OHD_3_ concentrations and demographic characteristics suggest that UVB exposure may play a greater role in maintaining circulating 25OHD_3_ concentrations than intrinsic factors such as sex, age, or reproductive status.

Comparisons of our black rhinoceroses 25OHD_3_ concentrations with previously published data suggest that individuals housed in North America exhibit lower circulating 25OHD_3_ than wild counterparts (Clauss *et al.*, 2002) and lower overall vitamin D compared to European managed populations (Bruins-van Sonsbeek and Corbee, 2024; Bruins-van Sonsbeek *et al.*, 2025) (Table 1). This finding is consistent with results from earlier North American studies (Olds *et al.*, 2018; Young *et al.*, 2019, unpublished work). However, direct comparisons are complicated by methodological differences in the majority of these earlier studies, including the form of vitamin D measured. Our use of LC–MS/MS enabled separate quantification of 25OHD_2_ and 25OHD_3_, whereas most earlier studies relied on radioimmunoassay (RIA) or enzyme-linked immunoassay, both of which measured total vitamin D (Olds *et al.*, 2018; Bruins-van Sonsbeek and Corbee, 2024; Bruins-van Sonsbeek *et al.*, 2025).

Contrary to age-related declines of circulating 25OHD_3_ described in some humans and rodent studies (Gallagher *et al.*, 2007; Ning *et al.*, 2016; Roizen *et al.*, 2018), age did not influence 25OHD_3_ concentrations in either white or black rhinoceroses. Notably, the highest summer 25OHD_3_ concentrations were observed in the two oldest individuals in the dataset, a 54-year-old white rhinoceros and a 32-year-old black rhinoceros. Although both individuals resided in a high irradiance zone (zone 5), age remained an insignificant factor even after accounting for irradiance zone. Similarly, in adult horses and other mammalian species, 25OHD_3_ concentrations are not influenced by age and vary primarily with environmental exposure (Pozza *et al.*, 2014; Handel *et al.*, 2016; Zhou *et al.*, 2019; Alizadeh *et al.*, 2022; Alemi *et al.*, 2025). These observations indicate that management-related factors, including outdoor access, shade availability, or daily husbandry schedules, may exert a stronger influence on vitamin D status than age-related physiological changes in managed rhinoceroses. Sex has also been identified as a potential contributor to vitamin D status in humans and some animal species, (Sharp *et al.*, 2015; Ning *et al.*, 2016; Feltrer-Rambaud *et al.*, 2023), but no sex-related differences were detected in the present study.

The lack of species differences in 25OHD_3_ concentrations, together with the high proportion of 25OHD_2_ values below detection limits, indicate a shared reliance on UVB radiation for maintaining vitamin D status in black and white rhinoceroses under managed care. This interpretation is supported by variation in 25OHD_3_ across solar irradiance zones and by consistent seasonal patterns observed in both species, pointing to endogenous synthesis as the primary contributor to circulating vitamin D, as reported in other African mammals (Young *et al.*, 2019, unpublished work; Callaby *et al.*, 2020; Bruins-van Sonsbeek and Corbee, 2024; Bruins-van Sonsbeek *et al.*, 2025). Although differences in natural foraging ecology could theoretically result in species-level variation in 25OHD_2_ (Boland *et al.*, 2003), diets under managed care are comparatively standardized across species (Clauss and Hatt, 2006; Sauspeter *et al.*, 2025), with reliance on commercially formulated pelleted feeds and supplemental browse likely reducing variation in dietary vitamin D status. Earlier studies raised concerns that black rhinoceroses might fail to maintain adequate circulating 25OHD concentrations during winter months, but those studies did not distinguish between 25OHD_2_ and 25OHD_3_, leaving the source of winter concentrations unresolved (Bruins-van Sonsbeek and Corbee, 2024; Bruins-van Sonsbeek *et al.*, 2025). In the present study, the consistently low 25OHD_2_ concentrations in both species, combined with quantifiable 25OHD_3_ concentrations in nearly all winter samples, indicate that black rhinoceroses are capable of maintaining endogenous 25OHD_3_ production during winter. Because the presumed half-life of 25OHD_3_ is two to three weeks (Batchelor and Compston, 1983), concentrations in early winter samples may reflect carryover from prior UVB exposure. However, 25OHD_3_ remained detectable in samples collected later in the winter (e.g. January).

Despite observed differences in 25OHD_3_ values among locations and seasons, concentrations did not vary substantially with white rhinoceroses reproductive status. If such a relationship existed, reproductively inactive individuals would be expected to exhibit lower 25OHD_3_ concentrations than actively cycling individuals. This hypothesis is supported by the presence of vitamin D receptors in reproductive tissues and evidence linking increases in vitamin D to reproductive performance, as well as to processes such as ovulation, follicular development, uterine receptivity, and implantation (Ota *et al.*, 2014; Handel *et al.*, 2016; Jukic *et al.*, 2018; Xu *et al.*, 2018; Jukic *et al.*, 2019; Zhou *et al.*, 2019; Xu *et al.*, 2021; Grzesiak *et al.*, 2022). We considered the possibility that differences between reproductive groups may have been obscured by pregnancy (*n* = 8–9) or lactation (*n* = 5–8), as heightened calcium demands during these states may reduce circulating 25OHD_3_ (Ardawi *et al.*, 1997). However, no differences were detected in 25OHD_3_ with pregnancy status, consistent with evidence indicating that circulating vitamin D typically remains stable during pregnancy and changes primarily in response to environmental factors such as sun exposure or supplementation (Ardawi *et al.*, 1997; Bärebring *et al.*, 2016; Goyal *et al.*, 2016). Early lactation in humans and dairy cattle has been shown to be associated with temporary decreases in 25OHD metabolites (Wilson *et al.*, 1990; Holcombe *et al.*, 2018), but exclusion of lactating individuals from our analyses did not alter the outcome.

There were elements of managed care not examined in this study that may influence 25OHD_2_ and 25OHD_3_ concentrations. For example, dietary 25OHD was not evaluated alongside serum values, and direct UVB exposure was not quantified. Prior work has demonstrated that rhinoceroses maintained entirely indoors exhibit remarkably low 25OHD_3_ concentrations despite supplementation (Young *et al.*, 2019, unpublished work), supporting a strong association between 25OHD_3_ and direct UVB exposure. Finally, time spent outdoors in different irradiance zones, or the total UVB exposure necessary for rhinoceros to maintain reference-range concentrations of 25OHD_3_, was not determined and would be valuable for guiding rhinoceros management recommendations.

This study provides the first circulating vitamin D assessment of managed white rhinoceroses and expands upon existing data for black rhinoceroses within North American institutions. Results demonstrate significant effects of season and location, specifically solar irradiance zone, but no detectable effects of age, sex, or reproductive status. Together, these findings offer an informative set of vitamin D reference values while providing strong evidence of UVB-induced 25OHD_3_ synthesis in North American black and white rhinoceroses. These data will support future targeted studies on environmental and management factors influencing vitamin D status and potentially related health conditions in rhinoceroses.

## Supplementary Material

Web_Material_coag062

## Data Availability

All relevant data can be made available upon request.
