# Supplementary material for: Vitamin D status in North American managed black rhinoceros (Diceros bicornis) and white rhinoceros (Ceratotherium simum)
Source: Conserv Physiol. 2026 Aug 25;14(1):coag062. doi: 10.1093/conphys/coag062 (PMC13503961; doi:10.1093/conphys/coag062)
Supplement: Supplementary file 1 [file web_material_coag062.zip › Vitamin_D_status_in_white_and_black_rhinoceros_SI.pdf]

VITAMIN D STATUS IN NORTH AMERICAN MANAGED BLACK (*DICEROS BICORNIS*) AND WHITE (*CERATOTHERIUM SIMUM*) RHINOCEROS

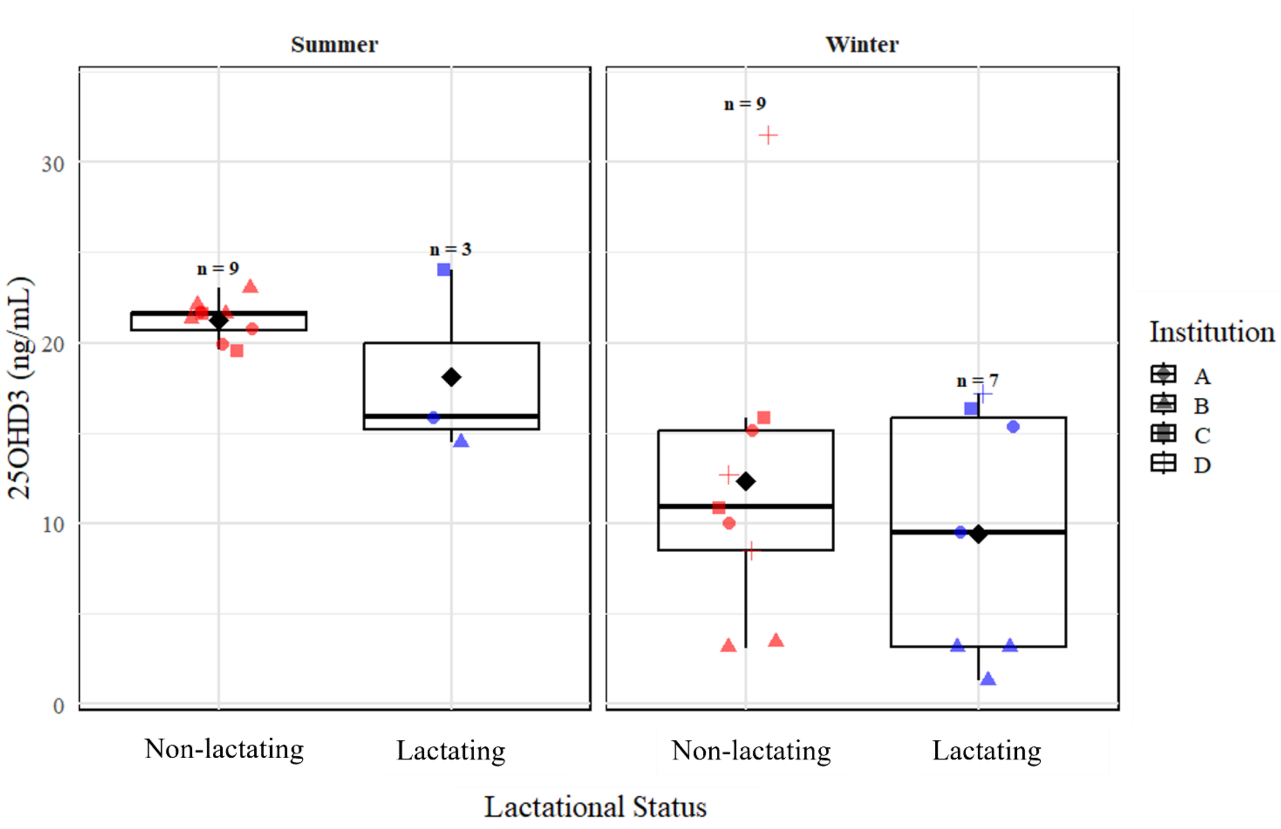

Figure S1. Circulating 25OHD3 concentrations in white (*Ceratotherium simum*) rhinoceros by season and lactational status from institutions housing both rhinos in both conditions. Lactational status was defined as the 12 months following the birth of a surviving calf. Statistical analyses were not performed due to limited sample sizes.
